# A hydraulic instability drives the cell death decision in the nematode germline

**DOI:** 10.1038/s41567-021-01235-x

**Published:** 2021-05-20

**Authors:** Nicolas T. Chartier, Arghyadip Mukherjee, Julia Pfanzelter, Sebastian Fürthauer, Ben T. Larson, Anatol W. Fritsch, Rana Amini, Moritz Kreysing, Frank Jülicher, Stephan W. Grill

**Affiliations:** 1 Biotechnology Center, TU Dresden, Dresden, Germany; 2 Max Planck Institute of Molecular Cell Biology and Genetics (MPI-CBG), Dresden, Germany; 3 Max Planck Institute for the Physics of Complex Systems (MPI-PKS), Dresden, Germany; 4 Center for Systems Biology Dresden, Dresden, Germany; 5 Center for Computational Biology, Flatiron Institute, New York, NY USA; 6 Department of Molecular and Cell Biology, University of California, Berkeley, CA USA; 7 Biophysics Graduate Group, University of California, Berkeley, CA USA; 8 Cluster of Excellence—Physics of Life, TU Dresden, Dresden, Germany

**Keywords:** Biological physics, Biophysics

## Abstract

Oocytes are large cells that develop into an embryo upon fertilization^1^. As interconnected germ cells mature into oocytes, some of them grow—typically at the expense of others that undergo cell death^2–4^. We present evidence that in the nematode *Caenorhabditis elegans*, this cell-fate decision is mechanical and related to tissue hydraulics. An analysis of germ cell volumes and material fluxes identifies a hydraulic instability that amplifies volume differences and causes some germ cells to grow and others to shrink, a phenomenon that is related to the two-balloon instability^5^. Shrinking germ cells are extruded and they die, as we demonstrate by artificially reducing germ cell volumes via thermoviscous pumping^6^. Our work reveals a hydraulic symmetry-breaking transition central to the decision between life and death in the nematode germline.

## Abstract (web summary)

During the early development of an organism, some cells are fated to grow while other seemingly healthy cells die. Experiments and theory now reveal that a hydraulic instability is the key to this decision.

## Main

**Fig. 1:**
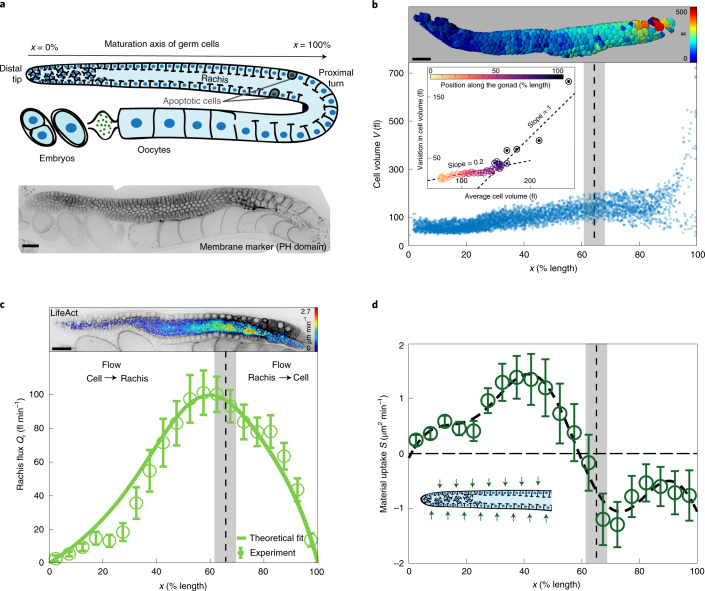
Volumes and fluxes in the *C. elegans* gonad. **a**, Top: schematic of an adult hermaphrodite gonad arm. Bottom: representative fluorescence image of a gonad expressing the membrane marker mCherry::PH(PLC1delta1). **b**, Top: germ cells colour-coded according to cell volume. Bottom: germ cell volume *V* along the gonad from distal tip (0% length) to proximal turn (100% length), for 5,265 germ cells from 18 gonad arms (bottom). Inset: s.d. plotted against the average of germ cell volumes determined at 40 colour-coded positions along the gonad. We observe two different relationships between s.d. and average, indicative of a transition between two growth modes. **c**, Top: cross-section of a gonad expressing LifeAct::mKate overlaid with colour-coded flow speeds as obtained by PIV (Supplementary Information). Bottom: cytoplasmic flux *Q*_r_ through the rachis as a function of position along the gonad. Open circles, *Q*_r_ determined from PIV speed distributions obtained from 10 gonad arms. Solid line, best parameter theory fit given the profile of material uptake *S* shown in **d**. **d**, Open circles denote material uptake *S* into the gonad from the outside, determined by volume conservation of rachis flux (**c**) and volume flux associated with germ cells moving from distal to proximal (Supplementary Fig. 1e). The thick dashed line shows a smoothened representation of the material uptake profile (Supplementary Information). Inset: green arrows indicate material uptake from the surrounding. The dashed vertical lines and grey boxes in **b**–**d** denote the position and associated confidence interval, respectively, where the distribution of germ cell volumes is no longer unimodal. Scale bars, 20 μm. Error bars indicate the error of the mean at 95% confidence.

The germline of the adult *Caenorhabditis elegans* hermaphrodite captures all the essential features to identify the mechanisms by which germ cells are selected to live or die. The nematode gonad is a tubular syncytium consisting of germ cells that surround a central cytoplasmic compartment called rachis, to which all the germ cells are connected via openings called rachis bridges^3,7–10^ (Fig. 1a). Germ cells originate in a mitotic zone from a pool of stem cells residing in the distal tip of each gonad arm, and undergo meiotic maturation as they move towards the proximal turn^3,11^. During this progression, some of the germ cells grow to become oocytes, while the rest shrink and die by physiological apoptosis^12^ (Fig. 1a). Although the core apoptotic machinery was shown to drive the final steps of cell death, the mechanisms that select and initiate apoptosis in individual germ cells are still unclear^13,14^.

Oocyte growth in *C. elegans* has been shown to rely on long-range cytoplasmic streaming^15,16^, but how and why germ cells shrink remains elusive. To identify a potential relationship between germ cell growth, shrinkage and apoptosis, we first set out to both quantify where germ cells grow along the gonad and characterize how this growth proceeds (Fig. 1b). Confocal imaging followed by three-dimensional (3D)-membrane-based segmentation of adult germlines expressing the membrane marker mCherry::PH(PLC1delta1) allowed us to measure individual germ cell volumes along the distal to proximal axis until the turn region (Supplementary Video 1). We find that germ cells near the distal tip have a volume of approximately 100 fl. As germ cells mature along the gonad, they first collectively grow in volume to approximately 150 fl (Supplementary Fig. 1a). Before the proximal turn, the variation in germ cell volumes increases drastically, and germ cells range from very small (~65 fl) to very large (~1,200 fl) sizes. While the distribution of germ cell volumes is unimodal in the distal region, it becomes bimodal close to the turn (Fig. 1b and Supplementary Fig. 1b). This suggests a transition from a homogeneous to a heterogeneous growth mode of germ cells along the gonad. To identify the precise location where this transition occurs, we investigated the average and standard deviation of germ cell volumes in different regions along the gonad (Fig. 1b, bottom inset). Both quantities appear to be linearly related but the associated slope changes sharply, which can be used to locate the transition zone to 65% ± 3.75% germline length. Two alternative methods for investigating the unimodality of distributions to identify the transition zone gave a similar result (Supplementary Information). Note that physiological apoptosis occurs proximal to this transition point, from about 70% to 90% germline length (Supplementary Fig. 1c).

Both homogeneous and heterogeneous modes of germ cell growth must rely on the addition of cytosolic volume. Germ cells can either grow by receiving material from the rachis inside or from the surrounding tissue outside, such as the intestine^17^. We set out to identify the two regions of growth from where the corresponding cytosolic volume is available. For this, we made use of the fact that cytosol is incompressible^18^ and determined the volume flux of cytoplasmic material through the rachis along the gonad *Q*_r_(*x*), where *x* denotes the position along the distal–proximal axis of the germline (Supplementary Information). In this one-dimensional (1D) representation, the flux balance at the steady state can be expressed as

1 $${\partial }_{x}{Q}_{\rm{r}}=J,$$

where *J*(*x*) denotes the germ-cell-to-rachis current. Hence, an increase in rachis flux *Q*_r_ along the gonad implies that germ cells contribute material to the rachis, while a decrease means that germ cells receive material from the rachis. We performed particle image velocimetry (PIV)^19^ on mid-plane confocal sections of the germline expressing LifeAct::mKate to determine a cytoplasmic velocity field inside the rachis, which we then used to infer the steady-state rachis flux (Fig. 1c, Supplementary Video 4 and Supplementary Information). We find that *Q*_r_ increases monotonically along the distal part of the gonad, peaks at approximately 60% germline length and decreases thereafter. Hence, *J* is positive before 60% germline length and germ cells donate material to the rachis, while *J* is negative and germ cells receive material from the rachis thereafter.

Interestingly, germ cells grow before 60% germline length (Figs. 1b and Supplementary Fig. 1a) despite losing cytoplasm to the rachis. This implies that they must be receiving material from the outside. We inferred the profile of material uptake *S*(*x*) (Fig. 1d) from the total flux balance at the steady state:

2 $${\partial }_{x}({Q}_{\rm{c}}+{Q}_{\rm{r}})=S,$$

where *Q*_c_(*x*) denotes the volume flux associated with germ cells moving from distal to proximal^11^ (Supplementary Information). We find that in the distal region and up to approximately 60% gonad length, material uptake *S* is positive and germ cells grow by receiving material from the outside. Material uptake becomes negative beyond 60%, indicating a loss of material to the outside, possibly via removal of apoptotic cells. We conclude that material associated with the homogeneous growth of germ cells comes from the outside, while the heterogeneous growth mode is associated with germ cells receiving cytoplasm from the rachis.

**Fig. 2:**
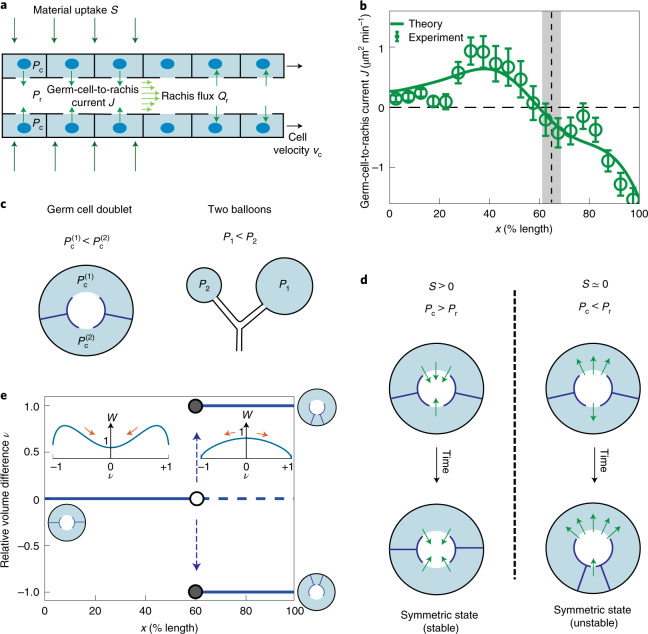
A theoretical model of germ cell and rachis fluxes reveals a hydraulic instability. **a**, Schematic of a 1D hydrodynamic model for pressures, material fluxes and volume exchange in the *C. elegans* gonad. *P*_c_ and *P*_r_ denote the pressure field in germ cells and rachis, respectively; *S*, profile of material uptake from the outside; *J*, germ-cell-to-rachis current associated with flows through the rachis bridges; *Q*_r_, rachis flux; and *v*_c_, germ cell velocities. **b**, Green open circles, estimated germ-cell-to-rachis current *J* along the gonad length; vertical dashed line and grey bar denote the region of transition between the growth modes of germ cells (Fig. 1b). Solid line, best parameter theory fit given the profile of material uptake *S* shown in Fig. 1d. **c**, Schematic of a germ cell doublet and two connected balloons depicting how a difference in volumes leads to difference in pressures. **d**, Evolution of small volume differences between coupled germ cells with time. The symmetric state (*ν* = 0) of equal germ cell volumes is unstable when the pressure in the rachis is higher than in germ cells (right). Here the initially larger cell grows at the expense of the smaller one. **e**, Relative volume difference *ν* in a cell doublet bifurcates around 60% germline length where material uptake *S* vanishes and pressure difference *P*_c_ – *P*_r_ changes sign. Insets illustrate cell configurations and corresponding effective potentials *W* as a function of *ν* normalized by its value at *ν* = 0 (Supplementary Information). Error bars indicate the error of the mean at 95% confidence.

The observed inversion of current *J* around 60% germline length implies an inversion of the pressure difference across the rachis bridges separating the germ cells from the rachis. To shed light on the underlying force balances, we construct a 1D physical model that relates pressure profiles to flows of germ cells and rachis cytoplasm as well as material exchange between germ cells and rachis (Fig. 2a and Supplementary Information). The germ-cell-to-rachis current is driven by differences in germ cell pressure *P*_c_(*x*) and rachis pressure *P*_r_(*x*), and it can be expressed as *J* = *α*(*P*_c_ – *P*_r_). Here *α* denotes an effective hydraulic conductivity of rachis bridges, which depends on rachis bridge radii. Using the profile of material uptake (Fig. 1d), this theory recapitulates rachis and germ cell fluxes (indicated by solid lines in Fig. 1c and Supplementary Fig. 1e) and predicts a profile of germ-cell-to-rachis current *J* that matches the experimental estimates (Fig. 2b). Consistent with the observation that the rachis flux peaks around 60% gonad length, this current changes sign at the same location (Fig. 2b). Because pressure differences drive the germ-cell-to-rachis current *J*, the pressure difference between the cells and rachis, *P*_c_ − *P*_r_, also changes sign at this location. Next, we investigated if this inversion of the pressure difference might be the key to understanding the transition from homogeneous to heterogeneous mode of germ cell growth. We note that the change in unimodal to bimodal volume distribution is indicative of instability in the germ cell volumes during growth. Similar instability arises when simultaneously blowing into two rubber balloons in an attempt to inflate them both. Here only one balloon inflates. Because the larger balloon can be inflated at lower pressures than the smaller one, the situation where both simultaneously inflate is mechanically unstable^20^.

Could such an instability also arise in the germline^21–27^? We consider the mechanics of a simplified configuration of two germ cells with volumes *V*_1_ and *V*_2_ that surround a common rachis to which they are connected by rachis bridges (Fig. 2c). We take into account force balances due to cortical tension and the fact that material uptake from the outside of each cell is proportional to the basal surface area exposed to the outside. Note that one germ cell can increase in size at the expense of the other. Furthermore, large germ cells tend to have larger rachis bridges than small germ cells (Supplementary Fig. 1f). With these components, we obtain an equation of motion for the relative volume difference *ν* = (*V*_2_ − *V*_1_)/(*V*_1_ + *V*_2_) (Supplementary Information):

3 $$\begin{array}{l}\frac{{\rm{d}}\nu }{{\rm{d}}t}=-\frac{S}{{A}_{{\rm{c}}}}\left(\lambda (\nu )+\nu \right)-{\alpha }_{0}\frac{{P}_{{\rm{c}}}-{P}_{{\rm{r}}}}{2{A}_{{\rm{c}}}}\nu (1-\nu )(1+\nu )\\\qquad\ +{\alpha }_{0}\frac{{{\Delta }}P(\nu )}{8{A}_{{\rm{c}}}}\left(1+6{\nu }^{2}+{\nu }^{4}\right),\end{array}$$

where *A*_c_ is the combined cross-sectional area of the two germ cells; *λ* is the relative difference in the basal surface area of the germ cells, which depends on *ν*; and *α*_0_ is the hydraulic conductivity. The average pressure between the two germ cells ${P}_{{\rm{c}}}^{(1)}$ and ${P}_{{\rm{c}}}^{(2)}$ can be expressed as ${P}_{{\rm{c}}}=({P}_{{\rm{c}}}^{(1)}+{P}_{{\rm{c}}}^{(2)})/2$. Pressure in the two germ cells can differ by ${{\Delta }}P={P}_{{\rm{c}}}^{(1)}-{P}_{{\rm{c}}}^{(2)}$. This pressure difference Δ*P* depends on the relative volume difference *ν* and is similar to the pressure difference between two balloons of different sizes (Fig. 2c). For two germ cells, Δ*P* ≈ *Tν*/*R*, where *T* is an effective cortical tension^9,15^ and *R* is the radius of the gonad (Supplementary Information). This pressure difference, therefore, tends to destabilize the symmetric configuration with equal germ cell volumes. Two additional contributions in equation (3) can stabilize the symmetric state. First, the effects of material uptake *S* > 0 are generally stabilizing. Second, the contributions from the difference in germ cell and rachis pressure become stabilizing when *P*_c_ > *P*_r_. Hence, material uptake in the distal region (*S* > 0) leads to a situation with *P*_c_ > *P*_r_, both of which stabilize the symmetric state of equal germ cell volume in this region. At around 60% gonad length, material uptake vanishes (*S* ≈ 0) leading to inversion of the pressure difference *P*_c_ − *P*_r_ at a point slightly proximal to the point at which the uptake vanishes (Figs. 1d and 2b). Beyond this point, the stabilizing effects are absent and the symmetric state is, therefore, unstable. As a result, small differences in germ cell volumes increase, leading to the growth of the larger germ cell at the expense of the smaller one until the small cell loses its cytoplasm at *ν* = ±1 (Fig. 2d). Equation (3) can be expressed in terms of an effective potential d*ν*/d*t* = –d*W*/d*ν* (Fig. 2e, inset). This effective potential *W* either has a single minimum at *ν* = 0 when the symmetric state with *ν* = 0 is stable or exhibits two minima at *ν* = ±1 corresponding to the two completely asymmetric configurations, while the symmetric state at *ν* = 0 is at a maximum. Hence, germ cells undergo homogeneous growth in the distal region before 60%, beyond which they undergo heterogeneous growth as is observed in this regime (beyond 65% gonad length; Fig. 1b). The transition between these two regimes is associated with a hydraulic instability, which is triggered by the loss of material uptake and the associated inversion of the pressure difference *P*_c_ − *P*_r_.

**Fig. 3:**
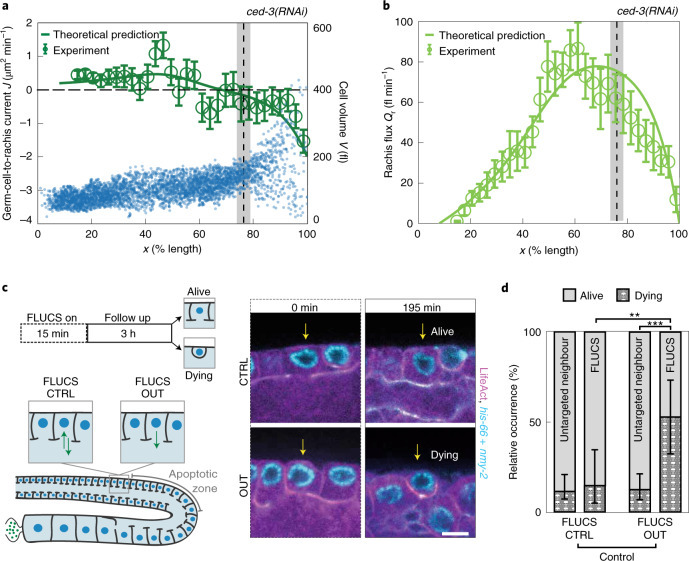
A hydraulic instability drives cell shrinkage that triggers cell death. **a**, Blue dots, cell volume *V* along the germline for 4,030 germ cells from 7 gonad arms where apoptosis was inhibited by *ced-3(RNAi)*. Vertical dashed lines and grey bars in **a** and **b** denote the region of transition between the growth modes of germ cells for *ced-3(RNAi)* (Fig. 1b). Green open circles, germ-cell-to-rachis current *J* in *ced-3(RNAi)* along the germline (Supplementary Information). Green line, profile of the germ-cell-to-rachis current *J* for *ced-3(RNAi)* as predicted by theory, using physical parameters and the profile of material uptake *S* obtained for the non-treated control (Supplementary Information, Fig. 1d and Supplementary Fig. 2d). **b**, Rachis flux *Q*_r_ along the gonad for *ced-3(RNAi)* obtained from 9 gonad arms (green open circles; Supplementary Information), together with the corresponding theoretical prediction (green line). **c**, FLUCS experiments. Left: schematic. Right: representative fluorescence images before and after bidirectional FLUCS as a control (CTRL; top) and unidirectional FLUCS (OUT; bottom) (magenta, LifeAct::mKate; cyan, GFP-*nmy-2* and Histone-Dendra). **d**, Relative occurrence of rachis bridge closure and rounding up within 3 h for cells treated with FLUCS CTRL or FLUCS OUT and their untargeted neighbours; **P < 0.01, ***P < 0.001. Only the FLUCS OUT-treated cells (right-most bar) displayed a significant increase in commencing apoptosis within 3 h. Scale bar, 5 μm. Error bars indicate the error of the mean at 95% confidence.

This hydraulic instability presents a possible mechanism by which germ cells become fated to die: generating a few large cells at the expense of smaller shrinking cells in a coarsening process. Apoptosis is then triggered in shrinking cells, which leads to their removal. However, an alternative scenario is that unknown molecular signals first induce apoptosis, which subsequently leads to the shrinkage of those cells fated to die. To test this alternative possibility, we inhibited the apoptosis of germ cells by RNA interference (RNAi)^28^ targeted against the caspase CED-3 (refs. ^12,13^) and evaluated if germ cells still shrink. We find that in the absence of apoptosis, germ cells are no longer removed^12^; however, we still observe that some cells in the proximal region shrink (blue dots in Fig. 3a). Similar to unperturbed conditions, *ced-3(RNAi)* gonads show a transition from a homogeneous to a heterogeneous mode of growth (grey bar in Fig. 3a); further, the position of this transition is close but a bit proximal to the location where both germ-cell-to-rachis current and pressure difference between the germ cells and rachis change sign (Fig. 3a), and where the rachis flux peaks (Fig. 3b). Together, this eliminates apoptosis as the cause of germ cell shrinkage and supports the idea that germ cell fate is determined by a hydraulic instability.

Our results suggest that a hydraulic instability generates large and small germ cells; the latter are eliminated by physiological apoptosis. This implies that increasing the number of small germ cells should lead to an increase in apoptotic germ cells. To test this, we depleted the small anillin isoform ANI-2 by RNAi, which is reported to affect the germline architecture and result in smaller germ cells^29^. Indeed, treatment with *ani-2(RNAi)* led to a higher fraction of proximal germ cells smaller than ~150 fl (Supplementary Fig. 2f,g) and a concomitant increase in the number of apoptotic cells^29,30^ (Supplementary Fig. 2h). Next, we set out to test if we can bias the outcome of this hydraulic decision-making process via direct mechanical manipulation. In particular, artificially reducing the volume of individual germ cells should increase their likelihood to undergo apoptosis. We tested this prediction by unidirectional thermoviscous pumping (focused-light-induced cytoplasmic streaming (FLUCS))^6^ for 15–20 min to pump germ cell cytoplasm out of individual germ cells through their rachis bridge, and monitoring the subsequent fate of the manipulated cells for 3 h (Fig. 3c). As a control, we performed bidirectional FLUCS by rapidly switching between pumping cytoplasm into and out of individual germ cells, with an overall similar dosage of laser light but without inducing a net flow (Supplementary Video 5). In the control scenario, 14.3% germ cells (3 out of 21) commence apoptosis within the following 3 h (Fig. 3d), as judged by a characteristic rounding up of apoptotic germ cells^12^. This number favourably compares with the rates of apoptosis in the unperturbed situation, since an apoptotic rate of 4–6% per hour (Supplementary Fig. 1c) leads to 11.5–17% germ cells commencing apoptosis within 3 h (Methods; note that we arrive at similar numbers when analysing neighbouring cells not subjected to FLUCS (Fig. 3d)). In contrast, 52.6% germ cells (10 out of 19) commence apoptosis within the three hours following unidirectional FLUCS (Fig. 3c,d and Supplementary Video 6). We conclude that a hydraulic manipulation to reduce the volume of individual germ cells results in an increased likelihood of commencing apoptosis. Together, this lends credence to the statement that the life and death decision in the gonad is of mechanical nature.

The hydraulic instability that we have discovered amplifies small differences in germ cell volumes and redistributes material from smaller to larger cells. This is consistent with observations in which larger oocytes continued to grow at the expense of smaller neighbours even after the blockage of rachis with an oil drop^15^. Because germ cell fate is determined by size, the mechanism selects for larger—and perhaps—fitter cells while making use of the resources of the dying ones^29,31^. The mechanism we have discovered here bases a cell-fate decision on a hydraulic instability, presenting a robust alternative to biochemical switches usually invoked in cellular decision-making processes^32–34^.

## Methods

### *C. elegans* strains

The following strains were used in this study:

**Table:** 

Strain name	Genotype
OD95	*unc-119(ed3) III; ltIs37 [Ppie-1::mCherry::his-58; unc-119(+)] IV; ltIs38 [Ppie-1::gfp::PH(PLC1delta1); unc-119(+)]*
UM208	*unc-119(ed3) III; ltIs81 [Ppie-1::gfp-TEV-Stag::ani-2; unc-119 (+)]; ltIs44 [Ppie-1::mCherry::PH(PLC1delta1); unc-119(+)] IV*
SWG007	*nmy-2(cp8 [nmy-2::GFP unc-119+]) I; gesIs001 [Pmex-5::LifeAct::mKate::nmy-2UTR, unc-119+]*
SWG016	*gesIs001 [Pmex-5::LifeAct::mKate::nmy-2UTR, unc-119+]; unc-119(ed3) III; opIs110 [lim-7p::YFP::actin + unc-119(+)] IV*.
SWG059	*gesIs001 [Pmex-5::LifeAct::mKate::nmy-2UTR, unc-119+]; unc-119(ed3) ruIs57 [pAZ147 pie-1p/GFP::C36E8.5] III*
SWG121	*tonSi1 [mex-5p::Dendra2::his-66::tbb-2 3'-UTR]; nmy-2(cp8 [nmy-2::GFP unc-119+]) I; gesIs001 [Pmex-5::LifeAct::mKate2::nmy-2UTR, unc-119+]*

### Confocal imaging of living germlines

Young adult worms (24 h post L4) were paralysed in 0.1% tetramisole (Sigma-Aldrich T1512) for 3 min on a cover slip precoated with 0.1% poly-l-lysine (Sigma-Aldrich P8920) and mounted on 2% agarose pads. Images were acquired with a spinning-disk confocal microscopy (Zeiss C-Apochromat, ×63/1.2NA, Yokogawa CSU-X1 scan head and Hamamatsu ORCA-Flash4.0 camera).

### Germ cell volume quantification

Confocal imaging was performed on UM208 young adult worms and 100 different *z* planes spaced 0.5 μm apart were acquired in 3 to 4 fields before being stitched together using Fiji’s pairwise stitching plugin. The 3D segmentation on the membrane marker was performed using Imaris Cell plugin (version 9.2).

### Rachis flow quantification

Confocal imaging was performed on SWG007 young adult worms and five different *z* planes spaced 2 μm apart were acquired every 10 s. To capture full gonads, 3 to 4 fields were acquired and stitched together using Fiji’s pairwise stitching plugin. The *z* stack corresponding to the central-most plane of the rachis was kept for analysis. Flow velocities were obtained by PIV tracking of LifeAct::mKate timelapse acquisitions^19^. PIV template size was 16 pixels corresponding to 1.695 μm. The *x* axis is defined as the distal to proximal axis. Due to geometric irregularities in the distal region of the rachis for *ced-3(RNAi)* germlines, we are only able to reliably identify steady velocity fields beyond 16% gonad length in the distal–proximal axis of these samples. The estimation of rachis flux *Q*_r_ from the velocity field is based on erosion-based segmentation to find the centreline and is described in Supplementary Information. Note that erosion-based segmentation fails near the proximal turn due to high curvatures. As a result, at the very proximal end and at 100% gonad length, the rachis flow into the turn is captured by the germ-cell-to-rachis current *J* rather than *Q*_r_. In our analysis, this results in the rachis flux *Q*_r_ to drop to nearly zero at 100% gonad length (Fig. 1c), slightly before the rachis flux actually drops to zero at the last oocyte^15^.

### Rachis opening perimeter

Confocal imaging was performed on UM208 young adult worms and 100 different *z* planes spaced 0.5 μm apart were acquired in 3 to 4 fields before stitching them together using Fiji’s pairwise stitching plugin. After maximal projections of the ANI-2::GFP slices corresponding to the upper half of the germline, a 2*σ* Gaussian blur filter was applied and the perimeter of each opening was quantified semi-automatically using Fiji’s wand tool. A conservative estimate for uncertainty is provided by the *z* spacing, which is 0.5 μm in this case.

### RNAi experiments

RNAi experiments were performed by feeding^28^. L4 worms were grown at 20 °C on feeding plates (NGM agar containing 1 mM isopropyl-β-D-thiogalactoside and 50 μg ml^–1^ ampicillin) for 48 h before imaging, else specified otherwise.

### FLUCS

Young adult worms were paralysed and mounted between a 3% agarose pad and a 18 mm × 18 mm coverslip (0.17 mm thickness). The sample was placed on a sapphire microscope slide equipped with Peltier cooling elements, sealed with dental silicone (Picodent twinsil, Picodent) and mounted on the FLUCS microscope stage^6^. The samples were imaged using ×60 water immersion objective (UPLSAPO, 1.2NA, W-IR coating, Olympus), D_2_O (Sigma-Aldrich) as the immersion fluid, 488 nm and 561 nm laser illumination, 1 × 1 binning and 30 s intervals at 20 *z* planes 1 μm apart (Olympus IX83, Yokogawa CSU-X1 scan head, Piezosystem Jena MIPOS 100, Andor iXon EMCCD DU-897 and Visitron VisiView software). Hydrodynamic flows were generated by scanning the 1,455 nm laser (Keopsys) at 1 kHz through the rachis opening. Custom-written LabVIEW software superimposed the scan path of the infrared laser with the fluorescence image of the camera. Following an approximately 15 min FLUCS treatment, the *z* stacks (1 μm spacing) were acquired every 15 min for 3 h. This allowed us to monitor the treated cell (FLUCS OUT, number of cells *n* = 19; FLUCS CTRL, *n* = 21) as well as its untreated neighbours (nearest, next-nearest and opposite; FLUCS OUT, *n* = 81; FLUCS CTRL, *n* = 94). Before and after FLUCS treatment, cell areas in each *z* plane were measured with Fiji’s freehand selection tool for integrating the volume of the treated cell (Supplementary Fig. 2f).

### Mitotic and apoptotic rates in the germline

SWG59 (for mitosis) or SWG16 (for apoptosis) young adult worms were paralysed in 0.1% tetramisole (Sigma-Aldrich T1512) for 3 min on a cover slip precoated with 0.1% poly-l-lysine (Sigma-Aldrich P8920) and mounted on 2% agarose pads. Images were acquired with a spinning-disk confocal microscopy (Zeiss C-Apochromat, ×63/1.2NA, Yokogawa CSU-X1 scan head and Hamamatsu ORCA-Flash4.0 camera) on 41 different *z* planes spaced 1 μm apart, recording one full stack every 30 s. After maximal projections of individual movies in Fiji, the number of metaphase spindles or each new engulfment event—together with their position relative to the distal tip or proximal turn—were manually measured. The frequency of mitotic or apoptotic events along the total amount of imaging time was then binned to obtain a probability of event per unit length of the germline (Supplementary Fig. 1c). From these rates, an estimate of cumulative probability can be deduced. For example, at position *x* of the gonad, considering that the rate of apoptosis per hour is *p*_a_, then the associated total probability of a cell to survive a period of 3 h is ${p}_{\rm{s}}={(1-{p}_{\rm{a}})}^{3}$. Hence, the estimated total cell death should be ~100 × (1 − *p*_s_)%.

### Cell number density in the germline

UM208 young adult worms (24 h post L4) were paralysed in 0.1% tetramisole (Sigma-Aldrich T1512) for 3 min on a cover slip precoated with 0.1% poly-l-lysine (Sigma-Aldrich P8920) and mounted on 2% agarose pads. Images were acquired with spinning-disk confocal microscopy (Zeiss C-Apochromat, ×63/1.2NA, Yokogawa CSU-X1 scan head and Hamamatsu ORCA-Flash4.0 camera) on 100 different *z* planes spaced 0.5 μm apart. To capture the full gonads, 3 to 4 fields were acquired and stitched together using Fiji’s pairwise stitching plugin. Curved gonads were straightened using a 300 pixels (32 μm) wide line on the *x*–*y* stacks using Fiji’s straighten tool and resliced along the *z* axis to get the cross-sections along the distal to proximal axis (semi-automatic using ‘sideviews’ Fiji macro). Cell numbers around the rachis were counted in 30 slices along the distal proximal axis and the average cell length along the distal proximal axis was estimated.

### Estimating interface curvature

UM208 young adult worms (24 h post L4) were paralysed in 0.1% tetramisole (Sigma-Aldrich T1512) for 3 min on a cover slip precoated with 0.1% poly-l-lysine (Sigma-Aldrich P8920) and mounted on 2% agarose pads. Images were acquired with spinning-disk confocal microscopy (Zeiss C-Apochromat, ×63/1.2NA, Yokogawa CSU-X1 scan head and Hamamatsu ORCA-Flash4.0 camera) on 100 different *z* planes spaced 0.5 μm apart. To capture the full gonads, 3 to 4 fields were acquired and stitched together using Fiji’s pairwise stitching plugin. Curved gonads were straightened using a 300 pixels (32 μm) wide line on the *x*–*y* stacks using Fiji’s straighten tool and resliced along the *z* axis to get the cross-sections along the distal to proximal axis (semi-automatic using ‘sideviews’ Fiji macro). For 1/100th of the cross-section slices, the curvature along all the cell–cell interfaces were calculated by fitting a 5 point B-spline curve using the Kappa plugin in Fiji. The average curvature (μm^−1^) at each cross-section was then plotted along the distal to proximal axis. A smooth curved surface of a cell of 10.0 μm height and 0.5 μm maximal deflection would correspond to a curvature of ~0.04 μm^−1^, larger than the estimated curvatures shown in Supplementary Fig. 4b.

### Reporting Summary

Further information on research design is available in the Nature Research Reporting Summary linked to this article.

## Online content

Any methods, additional references, Nature Research reporting summaries, source data, extended data, supplementary information, acknowledgements, peer review information; details of author contributions and competing interests; and statements of data and code availability are available at 10.1038/s41567-021-01235-x.

## Supplementary information

Supplementary Information Supplementary Information

Reporting Summary

Supplementary Video 1 The 3D fluorescent reconstruction and volumetric rendering of adult UM208 germline architecture. At the beginning of the video, the distal tip is to the left and the proximal turn to the right. Internal rachis lining is stained with *ani-2*::GFP (green channel) and germ cell membrane by mCherry::PH domain (red channel). The 3D rendering of individual segmented germ cells is colour-coded (blue, small; red, large). Finally, the white rendering depicts the structure of the rachis.

Supplementary Video 2 Maximum intensity projection of the proximal region of an adult SWG007 germline with two cells rounding up and closing their rachis bridge (white arrows). Left showing *nmy-2* as a single channel and right showing *nmy-2* in green and LifeAct::mKate in purple.

Supplementary Video 3 Maximum intensity projection of the distal region of an adult SWG59 germline showing mitotic spindle formation and division.

Supplementary Video 4 Central cross-section of an adult SWG007 germline. Cytoplasmic flows through the rachis are visible using LifeAct::mKate.

Supplementary Video 5 Central cross-section of an adult SWG121 germline. The white arrow indicates the manipulated cell with FLUCS CTRL, in which the rachis bridge remains open. Magenta, LifeAct::mKate; cyan, *nmy-2*::GFP and Dendra::*his-66*.

Supplementary Video 6 Central cross-section of an adult SWG121 germline. The white arrow indicates the manipulated cell with FLUCS OUT, which closes the rachis and individualizes during the course of the video. Magenta, LifeAct::mKate; cyan, *nmy-2*::GFP and Dendra::*his-66*.

## Figures and Tables

**Extended Data Fig. 1:**
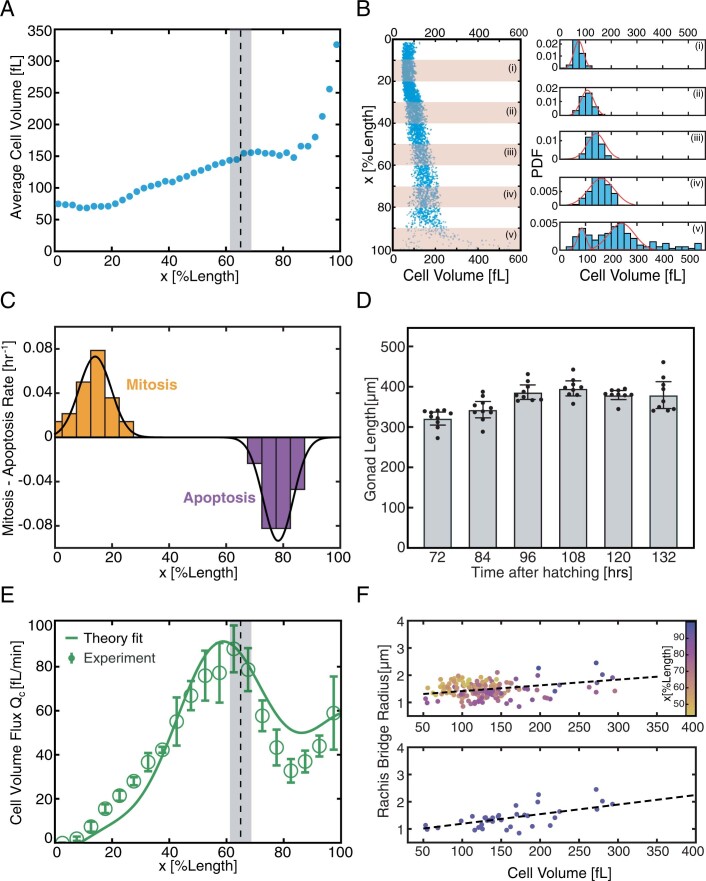
Volumes and fluxes in nematode germline. **A**, Average volume of germ cells along the distal to proximal axis (18 germlines, bin size 2.5 % gonad length). Vertical dashed line and grey region indicate the transition region between growth modes (Fig. 1b). **B**, Transition from a uni-modal to a bimodal distribution of germ cell volumes (left: blue dots, individual germ cell volumes as in Fig. 1b). Histograms of germ cell volumes in five different regions along the gonad (right). The distribution of germ cell volumes is unimodal (Gaussian fits in (i)-(iv)) in the distal region and bimodal in the proximal region (superposition of two Gaussian’s in (v)). **C**, Rates of mitotic (positive) and apoptotic events (negative) along germline length, analysed from 6 gonads for 30 mitotic events (Supplementary Video 2), and 4 gonads for 20 apoptotic events (Supplementary Video 3, see supplementary text). **D**, Gonad length as a function of time post hatching determined in at least nine worms. The adult germline length remains approximately constant. **E**, Green open circles, cell volume flux Q_c_ along the gonad. This flux peaks around 60% gonad length and decreases to a finite value, indicating outflow of cells at the proximal turn. Grey regions indicate the transition between growth modes (Fig. 1b). Green line, theory fit (see supplementary text). **F**, Correlation of rachis bridge radii with cell volumes. Top, rachis bridge radius plotted against cell volumes for individual germ cells along the gonad (color denotes position x). Rachis bridge radii correlate with germ cell volumes with a Pearson coefficient of 0.43. Bottom, restricting the analysis to the proximal regime with a broader distribution of germ cell volumes (beyond 75% gonad length) reveals a larger degree of correlation with a Person coefficient of 0.75. Errorbars indicate the error of the mean at 95% confidence.

**Extended Data Fig. 2:**
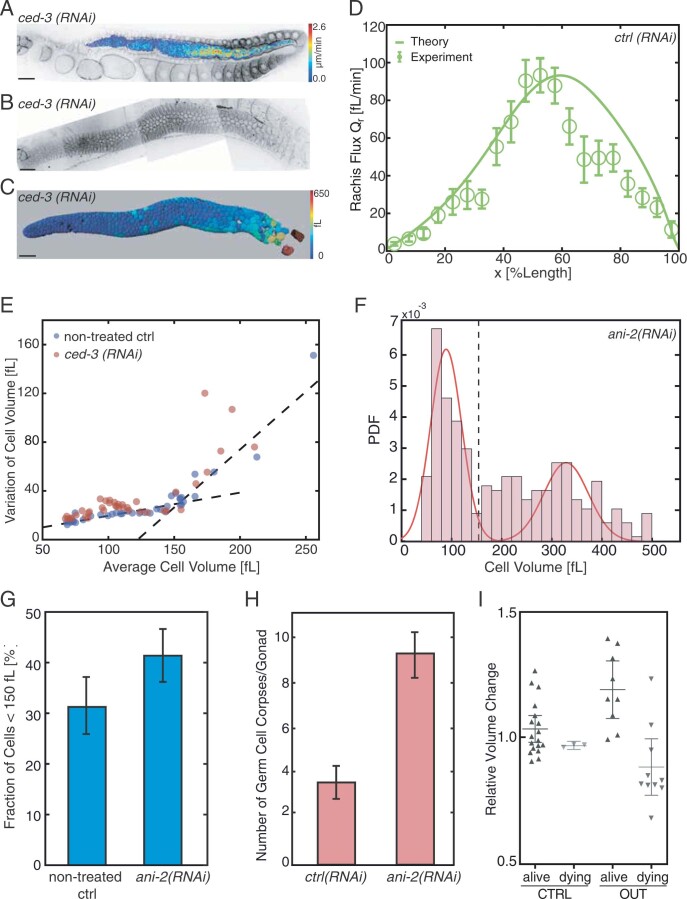
Genetic and physical perturbations of germ cells. A-C, Representative *ced-3(RNAi)* gonads. A, PIV on-axis speed (color-code) overlaid on a central cross section of a gonad expressing LifeAct::mKate treated with *ced-3(RNAi)*. B, Maximum intensity projection of an adult gonad treated with *ced- 3(RNAi)*, where the cell membrane expressing marker mCherry::PH-domain was imaged. C, 3D rendering of a representative *ced-3(RNAi)* germline where germ cells were segmented. Color code indicates germ cell volume. D, Open circles, rachis flux Q_r_ for the *L4440 control RNAi* from 10 gonad arms. Solid line, theory fit (see Supplementary Information). E, Standard deviation of cell volumes plotted against average cell volume for non-RNAi and *ced-3(RNAi)* germlines. F, Distribution of germ cell volumes located between 85%-100% gonad length treated with *ani-2(RNAi)* for 24 hours; solid curves indicate superposition of two Gaussian distributions; vertical dash line indicates crossover volume of 150 fL. G, Fraction of germ cells smaller than 150 fL located at proximal end (85%-100% gonad length) for non-treated control and *ani-2(RNAi)* condition. H, Number of germ cell corpses per gonad arm under *L4440 control(RNAi) (n=2)* and *ani-2(RNAi)* treatment *(n=3)*, identified by characteristic apoptotic rounding (see Supplementary Fig. 3). I, Relative volume change of germ cells during the approximately 15 min of thermoviscous pumping, for the FLUCS CTRL and FLUCS OUT experiment (Fig. 3c). Scale bars, 20 *μ*m. Error bars represent error of the mean at 95% confidence.

**Extended Data Fig. 3:**
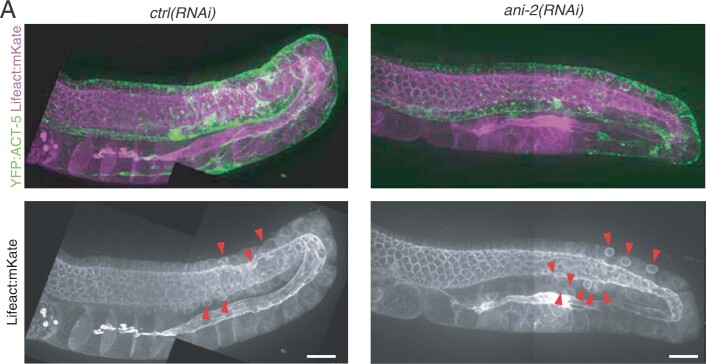
Apoptotic rounding of germ cells in non-treated and *ani-2(RNAi)* treated germlines. **A**, Maximum intensity projections of representative adult gonad (SWG016) treated with *L4440 control(RNAi)* (left panels) or *ani-2(RNAi)* (right panels) for 24 hours, showing actin in the germ cells (magenta) and in the surrounding sheath cells (green) engulfing the dying germ cells. Lower panels show actin in germ cells. Red arrows indicate germ cells with characteristic features of apoptotic rounding. Scale bars, 20 *μ*m.

**Extended Data Fig. 4:**
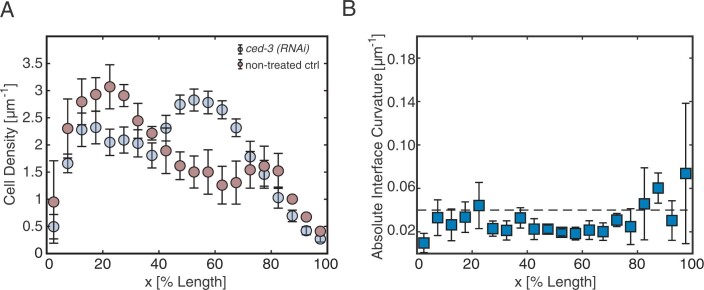
Cell density and interface curvature along the germline. **A**, Linear germ cell density profiles are shown for non-treated (n=11) and *ced- 3(RNAi)* treated (n=5) adult gonads. **B**, Spatial profile of mean absolute curvatures are shown (242 interfaces). Dashed line indicates curvature corresponding to a 0.5*μ*m central deflection of a 10*μ*m interface, below which we define the curvatures to be negligible (see Methods). Error bars represent error of the mean at 95% confidence.

## Data Availability

All data generated or analysed in this study are available from the corresponding authors upon reasonable request.

## Code availability

Codes to analyse the data and perform numeric calculations are available from the corresponding authors upon reasonable request.
