# Supplementary material for: A hydraulic instability drives the cell death decision in the nematode germline
Source: Nat Phys. 2021 May 20;17(8):920–5. doi: 10.1038/s41567-021-01235-x (PMC8548275; doi:10.1038/s41567-021-01235-x)
Supplement: Supplementary file 1 — Supplementary Information [file 41567_2021_1235_MOESM1_ESM.pdf]

nature portfolio

https://doi.org/10.1038/s41567-021-01235-x

Supplementary information

A hydraulic instability drives the cell death decision in the nematode germline

In the format provided by the authors and unedited

Supplementary Information

One-dimensional model of germline hydraulics

Volume conservation and force balance

Here we present the theoretical framework to discuss the hydraulics of the germline. We consider a one-dimensional representation of the gonad with positions $x \in [0, L]$ along the distal-proximal axis, where L is the gonad-length (Fig. SN1A below) which is typically $\sim 400\ \mu m$ (Extended Fig. S1D). We first consider volume conservation according to

$$\partial_t A_c + \partial_x(v_c A_c) = S - J \quad (1)$$

$$\partial_t A_r + \partial_x(v_r A_r) = J \quad . \quad (2)$$

Here A_c and A_r denote the cross-sectional areas that correspond to the germ cells and the rachis, respectively. Note that volume conservation implies flux balance at steady state. The average velocities of rachis cytoplasm and cells are denoted v_r and v_c, respectively. Material uptake from outside into cells is denoted S, and J signifies the germ cell to rachis current. This current is driven by the difference in cell pressure P_c and rachis pressure P_r, and given by

$$J = \alpha(P_c - P_r) \quad . \quad (3)$$

Here, $\alpha \simeq r_h^3/(3 l_c \eta)$ is a hydraulic conductivity of the rachis bridges, η denotes fluid viscosity, r_h is the radius of rachis bridges and l_c is the average cell length along the distal-proximal axis. The dependence of hydraulic conductivity α on the rachis bridge radius r_h results from pressure driven viscous flows through a circular orifice (Sampson flow)[1, 2]. The force balance in germ cells and rachis can be expressed according to

$$-\partial_x P_c = \gamma_o v_c + \gamma_r(v_c - v_r) \quad (4)$$

$$-\partial_x P_r = \gamma_r(v_r - v_c) \quad , \quad (5)$$

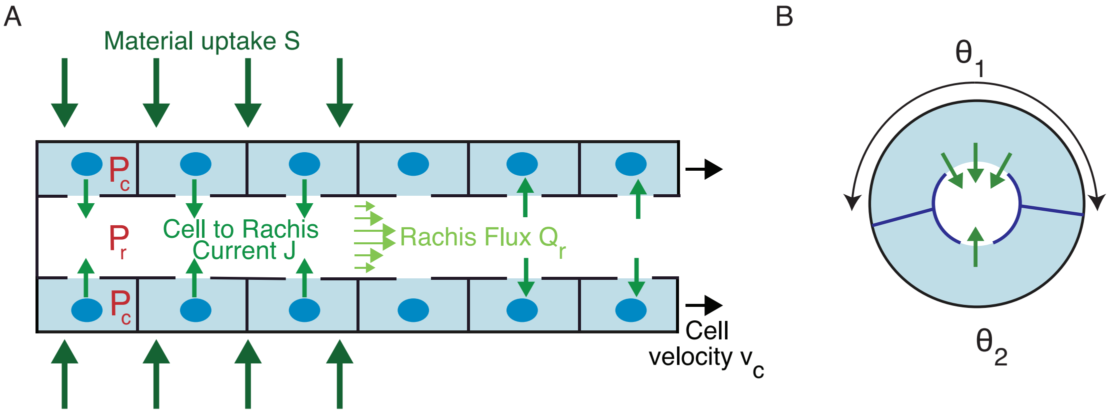

Fig. SN1: **A**, Schematic of hydrodynamic fluxes in the gonad and corresponding physical variables. **B**, The amount of material exchange between germ cells and rachis (green arrows) depends on their respective azimuthal angle θ.

where γ_o is a coefficient of friction between germ cells and the outside, and γ_r is an effective friction coefficient that describes frictional interactions between germ cells and the rachis fluid. These equations are supplemented by boundary conditions. The velocities of both germ cells as well as rachis fluid at the distal tip ($x = 0$) vanish: $v_c(0) = 0$ and $v_r(0) = 0$. At the proximal end ($x = L$), we have $v_r(L) = 0$. Note that volume conservation implies that $A_c v_c(L) = \int S dx$, i.e. the volume flux leaving the system at the proximal end $A_c v_c(L)$ equals the total material uptake $\int S dx$ from the outside. For this one dimensional description to accurately capture the physical states of the gonad, it is implicitly assumed that neighboring cells at a given location x have equivalent pressures. Pressure differences lead to curved cell-cell interfaces. Hence we evaluate if this criteria of force balance between neighboring cells is satisfied experimentally by estimating the curvature of cell-cell interfaces as a proxy for pressure differences among neighbors. We quantified the average absolute curvature of intra-cellular interfaces along the gonad, and found that cell-cell interfaces have negligible curvature ($\lesssim 0.02 \mu m^{-1}$) everywhere except for the very proximal region (see Supplementary Figure. S4B). We conclude that neighboring cells have equivalent pressures prior to $\sim 80\%$ gonad length.

Simple solutions for pressure and velocity profiles

For simplicity we consider the scenario where the areas A_c and A_r are constant along the gonad. In this case volume balance in steady state reads

$$\partial_x Q_c = S - \alpha(P_c - P_r) \tag{6}$$

$$\partial_x Q_r = \alpha(P_c - P_r) \quad , \tag{7}$$

where $Q_c = A_c v_c$ and $Q_r = A_r v_r$ are the cell and rachis volume fluxes, respectively. Combining these equations with the force-balance equations (Eqs. 4, 5) we obtain the velocity profiles which read

$$\partial_x^2 Q_c = \partial_x S + \frac{\alpha(\gamma_o + 2\gamma_r)}{A_c} Q_c - \frac{2\alpha\gamma_r}{A_r} Q_r \tag{8}$$

$$\partial_x^2 Q_r = -\frac{\alpha(\gamma_o + 2\gamma_r)}{A_c} Q_c + \frac{2\alpha\gamma_r}{A_r} Q_r \quad . \tag{9}$$

In redefined nondimensional units we obtain

$$\partial_x^2 Q_c = \partial_x S + \beta_c Q_c - \beta_r Q_r \tag{10}$$

$$\partial_x^2 Q_r = -\beta_c Q_c + \beta_r Q_r \quad . \tag{11}$$

Here $\beta_c = \alpha(\gamma_o + 2\gamma_r)/A_c$ and $\beta_r = 2\alpha\gamma_r/A_r$ are effective coefficients. We solve equations 10 and 11 for a given profile of material uptake $S(x)$ and the boundary conditions $Q_c(0) = Q_r(0) = Q_r(L) = 0$ employing a collocation method using the three-stage Lobatto IIIa formula. To specify the profile of material uptake we fit the function $S(x) = a_0 + \sum_{n=1}^{3}(a_n \cos(nx/L) + b_n \sin(nx/L)$ to the experimentally inferred profile of material uptake S shown in Fig. 1D. This provides us with a smooth and continuous representation $S(x)$ of the data (Fig.1D, dashed line). We then fit numerically determined solutions of Eqs. 10 and 11 to the profile of cell and rachis flows Q_c and Q_r using β_c and β_r as fit parameters (Figs. 1C, S1C). The effective

fit-parameters β_c & β_r define two length-scales $\beta_{c,r}^{-1/2}$. We represent the values obtained by the fitting procedure in terms of the gonad length L.

Fit parameter	value (L^{-2})	95% CI (L^{-2})
β_c	86.3	± 14.7
β_r	61.4	± 16.4

Both β_c and β_r are comparable in magnitude. This would be expected as the cell volume flux and rachis flux show comparable magnitudes and also range through comparable lengthscales.

Analysis of the RNAi condition where apoptosis is inhibited

To analyse the rachis flux profile under *ced-3(RNAi)*, we first analyse the rachis flux profile in the control condition *L4440* investigated 96 hours after hatching, which is 24hrs later than experiments in the non-RNAi condition shown in Figs. 1 and 2. We fit solutions to Eqs. 10 and 11 using the values of β_c and β_r obtained for the non-RNAi condition. We use a material uptake profile $\beta_s S(x)$, where $S(x)$ is the material uptake profile from Fig.1D, and β_s is a dimensionless factor scaling the amplitude of $S(x)$. Through this we account for changes in amplitude but not profile of material uptake between the two different time points of investigation 24 hours apart. The corresponding fit for L4440 control condition is shown in Fig. S2D.

Based on the now determined values for β_c, β_r and the material uptake profile $\beta_s S(x)$, we can predict the rachis flux profile for *ced-3(RNAi)* where apoptosis is suppressed. The observation that no cells are extruded suggests that material only leaves the system at the proximal end. We therefore postulate that the material uptake profile S_p for *ced-3(RNAi)* can be approximated as

$$S_p(x) = \begin{cases} \beta_s S(x) & , \forall x : S(x) > 0 \\ 0 & , \forall x : S(x) < 0 \end{cases} \quad . \tag{12}$$

Using this profile, we obtain the predicted rachis flux profile for *ced-3(RNAi)* shown in Fig. 2B. The agreement between prediction and data supports the idea that the suppression of apopto-

sis and the resultant inhibition of cell extrusion eliminates material leakage, but leaves other features of the system unchanged.

Azimuthal mechanical stability of a cell pair

The change in sign of the germ cell to rachis current J at $x \simeq 0.6\,L$ implies an inversion of the pressure difference $P_c - P_r$. We now discuss how the stability of a pair of cells located along the gonad at position x depends on the pressure profiles in the gonad. We consider two cells with total cross-section area $A_c = A_1 + A_2$ covering the azimuthal angles θ_1 and θ_2, respectively with $\theta_1 + \theta_2 = 2\pi$ (see Fig. SN2). The basal surface area of cell $i = 1, 2$ is $A_b^{(i)} = \theta_i R_b l_c$, where R_b is the radius of the germline. The volume of cell $i = 1, 2$ is given by $V_i = A_i l_c$, where l_c is the distal-proximal cell length. Because of material exchange the cell volume obeys

$$\frac{dV_i}{dt} = S_i l_c - J_i l_c \quad , \tag{13}$$

where $S_i l_c$ is the rate of volume uptake of cell i, and $J_i l_c$ denotes the rate of volume loss from cell i to rachis, with $S_i = S\theta_i/(2\pi)$ and $J_i = \alpha_i(P_c^{(i)} - P_r)$. We have introduced $\alpha_i \simeq r_{h,i}^3/(3 l_c \eta)$, where the rachis bridge radius of cell i is given by $r_{h,i}$. To determine the force balances governing the volume changes we use a simplified mechanical model of the germ-cell doublet, which can be captured in terms of a work function, typically expressed as,

$$E = \sum_\kappa T_\kappa A_\kappa \quad , \tag{14}$$

where T_κ denotes the cortical tension of the surface κ with area A_κ. We parametrize the cell geometry by the rachis radii R_i corresponding to cells $i = 1, 2$ as well as the relative difference in basal surface area $\lambda = (A_b^{(1)} - A_b^{(2)})/(A_b^{(1)} + A_b^{(2)})$ (see Fig. SN2A). For a germ-cell doublet we then write

$$E(R_1, R_2, \lambda) = \pi T_F \left(\frac{(1+\lambda)}{2}(R_b^2 - R_1^2) + \frac{(1-\lambda)}{2}(R_b^2 - R_2^2) \right) l_c + (\pi T_R(1+\lambda) - T_S) R_1 l_c$$
$$+ (\pi T_R(1-\lambda) - T_S) R_2 l_c + 2 T_S R_b l_c \quad .$$

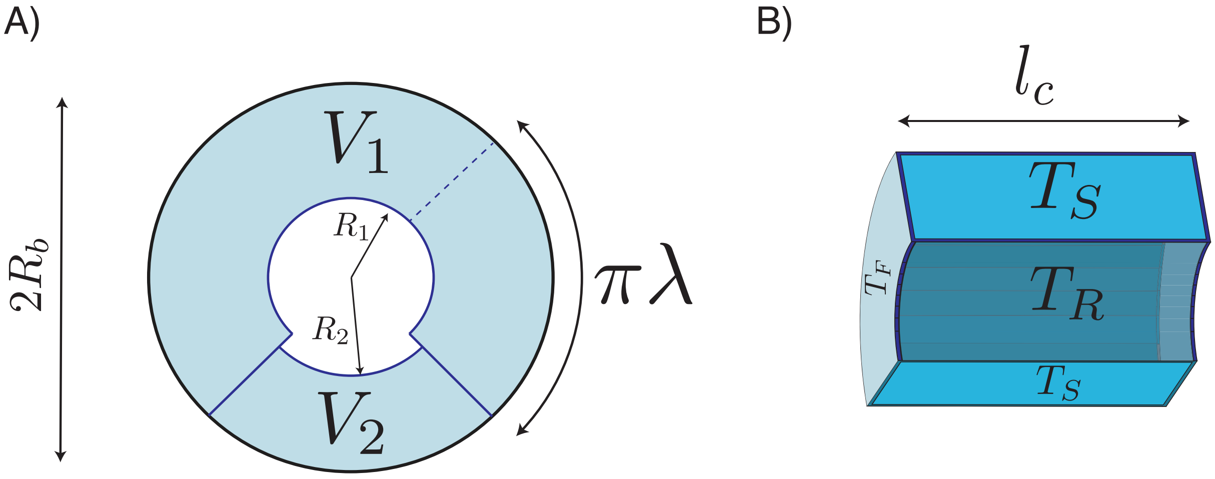

Fig. SN2: **A)** Schematic of the general doublet model. The geometric variables are depicted. **B)** Cortical tensions of the corresponding surfaces are schematically depicted.

The cell surface tensions T_F, T_S and T_R are associated with surfaces as depicted in Fig. SN2B. Here we ignore the tension of the basal/outer surface as it does not contribute to the radial force balance of R_1 and R_2. The volumes of the cells are given by $V_1 = \pi(1+\lambda)(R_b^2 - R_1^2)l_c/2$ and $V_2 = \pi(1-\lambda)(R_b^2 - R_2^2)l_c/2$, and the corresponding cross-sectional areas are $A_1 = \pi(1+\lambda)(R_b^2 - R_1^2)/2$ and $A_2 = \pi(1-\lambda)(R_b^2 - R_2^2)/2$. We use Langrange multipliers P_1 and P_2 to impose cell volumes V_1 and V_2 and the Lagrange multiplier Σ to impose the total cross-sectional area $A_c = A_1 + A_2$. Therefore we minimize the function,

$$F = E + P_1 V_1 + P_2 V_2 + \Sigma A_c \quad , \tag{15}$$

where P_i are the pressure differences $P_i = P_r - P_c^{(i)}$ for cell $i = 1, 2$. Mechanical equilibrium implies $\partial F/\partial R_i = 0$ and $\partial F/\partial \lambda = 0$, which yield expressions for the pressures differences P_i as well as a force balance equation according to

$$P_1 = \frac{T_R}{R_1} - \frac{T_S}{\pi(1+\lambda)R_1} - \frac{T_F + \Sigma}{l_c} \tag{16}$$

$$P_2 = \frac{T_R}{R_2} - \frac{T_S}{\pi(1-\lambda)R_2} - \frac{T_F + \Sigma}{l_c} \tag{17}$$

$$T_S(R_2^2 - R_1^2) + 2T_R(R_1 - R_2)l_c = -P_1(R_b^2 - R_1^2)l_c + P_2(R_b^2 - R_2^2)l_c \quad . \tag{18}$$

Using the expressions for the pressure differences P_i into Eq. 18 we obtain

$$\pi T_R(R_2-R_1)(R_b-R_1R_2)(1-\lambda)^2 = T_S R_2(R_b^2-R_1^2)(1-\lambda)-T_S R_1(R_b^2-R_2^2)(1+\lambda) \quad . \tag{19}$$

The difference in pressure $\Delta P = P_2 - P_1$ is given by,

$$\Delta P = \frac{T_R}{R_2} - \frac{T_R}{R_1} + \frac{T_S}{\pi}\left(\frac{1}{R_1(1+\lambda)} - \frac{1}{R_2(1-\lambda)}\right) \quad . \tag{20}$$

The volume balance for the two cells can be written as

$$\frac{dV_1}{dt} = S\left(\frac{1+\lambda}{2}\right) l_c + \alpha_1 \left(P_r - P_c - \frac{\Delta P}{2}\right) l_c \tag{21}$$

$$\frac{dV_2}{dt} = S\left(\frac{1-\lambda}{2}\right) l_c + \alpha_2 \left(P_r - P_c + \frac{\Delta P}{2}\right) l_c \quad . \tag{22}$$

Note that the pressure difference ΔP can also be expressed as $\Delta P = P_c^{(1)} - P_c^{(2)}$. The balance of total volume $V = V_1 + V_2$ with $dV/dt = A_c dl_c/dt$ is given by

$$\frac{1}{V}\frac{dV}{dt} = \frac{S}{A_c} + \frac{\alpha_1+\alpha_2}{A_c}(P_r - P_c) + \frac{\alpha_2-\alpha_1}{A_c}\frac{\Delta P}{2} \quad . \tag{23}$$

The difference in volumes $\Delta V = V_2 - V_1$ obeys

$$\frac{1}{V}\frac{d\Delta V}{dt} = -\frac{S}{A_c}\lambda + \frac{\alpha_2-\alpha_1}{A_c}(P_r - P_c) + \frac{\alpha_1+\alpha_2}{A_c}\frac{\Delta P}{2} \quad . \tag{24}$$

These expressions lead to the following equation of motion for the relative volume difference $\nu = (V_2 - V_1)/V$,

$$\begin{aligned}\frac{d\nu}{dt} = -\frac{S}{A_c}(\lambda+\nu) - \frac{P_c - P_r}{A_c}(\alpha_2 - \alpha_1 - \nu(\alpha_1+\alpha_2)) \\ + \frac{\Delta P}{2A_c}(\alpha_1+\alpha_2+\nu(\alpha_2-\alpha_1)) \quad .\end{aligned} \tag{25}$$

Here λ, α_i as well as ΔP are in general ν dependent. In particular we use $\alpha_1(\nu) = \alpha_0(1-\nu)^3/8$ and $\alpha_2(\nu) = \alpha_0(1+\nu)^3/8$, where $\alpha_0 = (m^3/3\eta)A_c^3 l_c^2$, which is based on the idea that the

rachis bridge radii depend on cell volume as $r_{h,i} \simeq mV_i$. The dynamics of the relative volume difference ν can be written as,

$$\frac{d\nu}{dt} = -\frac{S}{A_c}\left(\lambda(\nu) + \nu\right) - \alpha_0 \frac{P_c - P_r}{2A_c}\nu(1-\nu)(1+\nu) + \alpha_0 \frac{\Delta P(\nu)}{8A_c}\left(1 + 6\nu^2 + \nu^4\right) \quad . \tag{26}$$

The function $\lambda(\nu)$ and $\Delta P(\nu)$ can be obtained from force balance Eq.28 with imposed area A_c. The area constraint requires,

$$\lambda = \frac{2r_0^2 - r_1^2 - r_2^2}{r_1^2 - r_2^2} \tag{27}$$

where we have introduced nondimensional variables $r_i = R_i/R_b$ and $r_0^2 = 1 - A_c/(\pi R_b^2)$. The force balance 19 can be written as,

$$\pi(r_1 - r_2)(r_1 r_2 - 1)(1 - \lambda^2) - \sigma(r_1 + r_2)(r_1 r_2 - 1)\lambda + \sigma(r_1 - r_2)(1 + r_1 r_2) = 0 \quad , \tag{28}$$

where $\sigma = T_S/T_R$. Introducing the variables $A = r_1^2 + r_2^2$, $B = 2r_1 r_2$ and $A_0 = 2r_0^2$ and eliminating

$$\lambda = \frac{A_0 - A}{\sqrt{A^2 - B^2}} \tag{29}$$

in Eq.28, we obtain a quadratic equation for A given B, which reads

$$\begin{aligned} &\sigma B A^2 + \left(\frac{B}{2} - 1\right)\left(2A_0\pi - \sigma A_0 + \sigma B\right) A \\ &\qquad - \left(\frac{B}{2} - 1\right)\left(\pi(A_0^2 + B^2) + \sigma A_0 B\right) - \sigma\left(\frac{B}{2} + 1\right) B^2 = 0 \quad . \end{aligned} \tag{30}$$

From A and B we can determine,

$$r_1 = \frac{1}{2}\left(\sqrt{A + B} - \sqrt{A - B}\right) \tag{31}$$

$$r_2 = \frac{1}{2}\left(\sqrt{A + B} + \sqrt{A - B}\right) \tag{32}$$

$$\nu = \frac{A^2 - B^2 - (A_0 - A)(2 - A)}{(2 - A_0)\sqrt{A^2 - B^2}} \quad . \tag{33}$$

From these expressions one can calculate $d\nu/dt$ as a fucntion of ν given by Eq.26. Two examples are shown in Fig. SN3.

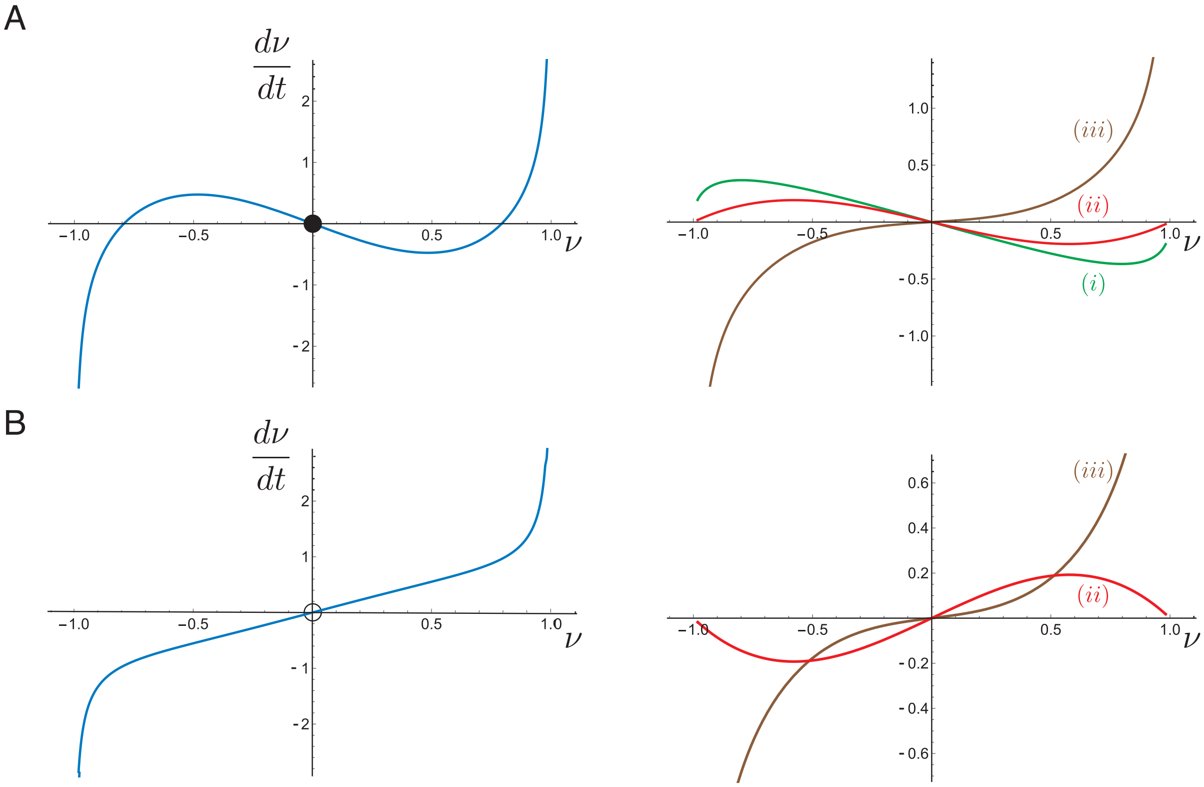

Fig. SN3: **A)** Left: Stability diagram of ν is shown for $S > 0$ and $P_c > P_r$. The symmetric state $\nu = 0$ is a stable fixed point and depicted with a solid circle. Right: Individual contributions from different terms in Eq.26 are shown for the the associated stability diagram: (i)(Green) ,(ii)(Red), (iii)(Brown) correspond to contributions due to material uptake S, pressure difference between germ cell to rachis $P_c - P_r$ and pressure difference between the germ cells ΔP . **B)** Left: Stability diagram of ν is shown for $S = 0$ and $P_c < P_r$. The symmetric state $\nu = 0$ is an unstable fixed point and depicted with an empty circle. Right: Individual contributions from different terms are shown for the the associated stability diagram.

In panel A of Fig. SN3 we consider a scenario akin to the distal region of the germline where material uptake $S > 0$ and $P_c > P_r$. In this scenario the symmetric state at $\nu = 0$ is a stable fixed point. While contributions from S and $P_c - P_r$ stabilize the symmetric state $\nu = 0$, the pressure difference between the two cells ΔP destablizes the symmetric state. In panel B of Fig. SN3 we consider $S = 0$ and $P_c < P_r$. As a result the stabilizing effect of S is absent and contributions from $P_c - P_r$ as well as ΔP make the symmetric state $\nu = 0$ unstable. The

disappearance of material uptake S as well as change of sign of $P_c - P_r$ leads to the change in stability of the symmetric state and we refer to this as the hydraulic instability.

This analysis can also be presented in terms of an effective potential W given by,

$$\frac{d\nu}{dt} = -\frac{dW}{d\nu} \tag{34}$$

The effective potentials W associated with stability diagrams in Fig. SN3 are depicted as insets of Fig. 2E, where we have normalized the potential by $W(\nu = 0)$.

Eq.26 can be linearized around the symmetric state $\nu = 0$ and is given by

$$\frac{d\nu}{dt} \simeq -\frac{\alpha_0}{A_c}\left(\frac{S}{\alpha_0}(1-b) + \frac{5}{8}(P_c - P_r) - \frac{\Delta P_0}{16}\right)\nu \quad . \tag{35}$$

Here the coefficients b and ΔP_0 are,

$$b = \left.\frac{d\lambda}{d\nu}\right|_{\nu=0} = \left(\frac{\pi}{\sigma} - \frac{1+r^2}{1-r^2}\right)\left(\frac{\pi}{\sigma} - 1\right)^{-1} \quad , \tag{36}$$

$$\Delta P_0 = \left.\frac{d\Delta P}{d\nu}\right|_{\nu=0} = \frac{2T_S}{\pi R_b r}\left(2 - \frac{2\sigma r^2}{(1-r^2)(\pi-\sigma)}\right) \quad , \tag{37}$$

where $r = (r_1 + r_2)/2$ and $r_2 - r_1 << r$. The symmetric state is stable iff

$$\frac{S}{\alpha_0}(1-b) + \frac{5}{8}(P_c - P_r) - \frac{\Delta P_0}{16} > 0 \quad . \tag{38}$$

We consider the case where $S(1-b)$ and ΔP_0 are positive everywhere. In the distal region, S is sufficiently large such that $S(1-b)/\alpha_0 - \Delta P_0/16 > 0$ and therefore the equally-sized cell state is stable. This is further ensured by $P_c > P_r$ in this region. When S vanishes near 60% germ line length, the hydraulic instability is driven by ΔP_0 and supported by the pressure difference $P_c - P_r$ becoming negative in this region (see Extended Data Fig. S4B). For $R_1 = R_2 = R$, the asymmetry can only arise in terms of the azimuthal angle and we find $\lambda = -\nu$. In this case the pressure difference (Eq.20) between the cells is given by,

$$\Delta P = \Delta P_1 \frac{2\nu}{1-\nu^2} \quad , \tag{39}$$

where $\Delta P_1 = T_S/\pi R$. Eq.26 then takes the following form,

$$\frac{d\nu}{dt} = -\alpha_0 \frac{P_c - P_r}{2A_c} \nu(1-\nu)(1+\nu) + \alpha_0 \frac{\Delta P_1}{4A_c} \frac{\nu}{1-\nu^2} \left(1 + 6\nu^2 + \nu^4\right) \quad . \tag{40}$$

Here the stabilising effect originating from S is absent but the destablising effect of ΔP persist. Then the onset of the hydraulic instability is driven by the inversion of pressure difference $P_c - P_r$, in the same way as described for Eq.26.

Identification of the transition point between the two growth modes

We plot the standard deviation of cell volumes against the mean of cell volumes for 40 spatial bins along the gonad (see inset Fig. 1B, Fig. S2E). We find that they follow a linear relationship in the distal region, which transitions to another linear relationship with higher slope towards the proximal end. We identify this crossover point by fitting linear curves to the first three and last three points and identifying their intersection at $\sim$ 150fL mean cell volume. We then calculate the Euclidean distance of each point from this intersection of two lines. To define a region of transition we then use a span of $1.5d_c$ where d_c is the distance of the closest point to the intersection of the two lines. We find that this crossover region lies between $61.25\% - 68.75\%$ gonad length in space along the distal to proximal axis in the unperturbed gonads and at $73.75\% - 78.75\%$ gonad length for *ced-3(RNAi)*. We use two additional metrics to evaluate the dispersion of the germ cell volume population, namely the Gini and the Hoover index[3]. These both show a monotonic increase beyond the above specified transition regions, respectively (see Fig. SN3 below).

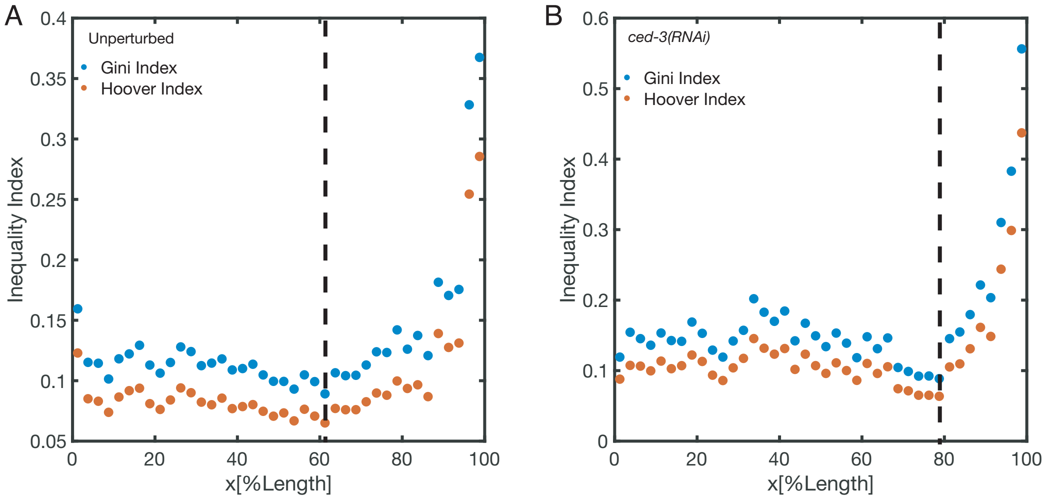

Figure. SN4: Gini (blue dots) and Hoover (orange dots) indices in (**A**) non-RNAi and (**B**) *ced-3(RNAi)* conditions. Vertical lines indicate the position of minima which are identical for both measures.

87 ## Determination of the rachis flux Q_r

Starting from the velocity field obtained by performing PIV (inset of Fig. 1C, Fig. S2A) on the midsection of the gonad. We acquire a centerline of the gonad by erosion based segmentation. At each PIV grid point we identify the velocity component tangential to this centerline. For each point along the centerline we pursue a cut orthogonal $(\perp)$ to the centerline and determine the tangential velocity components along this cut. We then fit a parabolic function to this orthogonal velocity profile using

$$v_\perp[r] = v_\perp^{max}\left(1 - \frac{r}{R_r}\right)^2 \quad , \tag{41}$$

using a hard tolerance criteria. Here, $v_\perp^{max}$ denotes the peak velocity, and R_r is the resultant rachis radius. We then integrate over this function to estimate the rachis flux Q_r at this centerline position according to

$$Q_r = \int d\phi \int_{-R_r}^{+R_r} v_\perp[r] r dr = \frac{v_\perp^{max}}{2}\pi R_r^2 \quad . \tag{42}$$

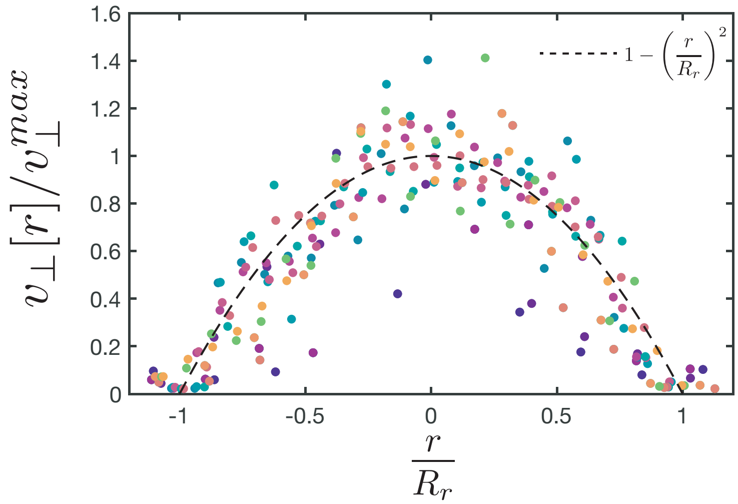

Figure. SN5: Example of 20 individual velocity profiles perpendicular to the centerline.

The average velocity of fluid through rachis $v_r = Q_r/A_r$ relates to the peak velocity for a parabolic flow as $v_r = v_{\perp}^{max}/2$. Note that erosion based segmentation is vulnerable to high curvatures and as a result near the turn the centerline does not imitate the turn. Hence the flow into the turn is treated as a radial flow orthogonal to the centerline and contributes to the germ cell to rachis current J rather than to Q_r.

Determination of the germ cell volume flux Q_c

We start out by the assumption of conservation of cell density n (see Fig. S4 A) in the gonad according to

$$\partial_t n + \partial_x(n v_c) = (k_d - k_a)n \quad , \tag{43}$$

where nv_c is the cell density flux, k_d is the rate of mitosis and hence the birth rate, and k_a is the rate of apoptosis. We assume steady-state (Fig. S1D) and integrate over birth and death rates (Fig. S1C) and find

$$v_c[x] = \frac{n[0]}{n[x]} v_c[0] + \frac{1}{n[x]} \int_o^x \left(k_d[x'] - k_a[x']\right) n[x'] dx' \quad . \tag{44}$$

Cell velocities at the distal-end are zero ($v_c[0] = 0$). Combining with the cross-sectional areas of the gonad, we estimate the volume flux due to cell movement (Fig. S1E) as the product of cell velocity and cellular area ($Q_c = A_c v_c$).

Determination of the profile of material uptake S from the surrounding

To infer the material uptake profile S of the germline from the surrounding environment, we make use of volume conservation and steady-state and obtain

$$\partial_x (Q_c + Q_r) = S \quad . \tag{45}$$

Therefore, the spatial gradient of the total volume flux (i.e. the rachis flux Q_r plus the cell volume flux Q_c) informs us of material uptake at a given location. We use a 4-point central difference method to estimate this gradient and the corresponding 95% confidence intervals (Fig. 1D).

References

[1] Sampson, R. A. On stokes's current function. *Philosophical Transactions of the Royal Society of London.(A.)* 449–518 (1891).

[2] Happel, J. & Brenner, H. *Low Reynolds number hydrodynamics: with special applications to particulate media*, vol. 1 (Springer Science & Business Media, 2012).

[3] Coulter, P. B. *Measuring inequality: A methodological handbook* (Routledge, 2019).
